# *Wnt7A* is a putative prognostic and chemosensitivity marker in human malignant pleural mesothelioma

**DOI:** 10.3892/or.2015.3771

**Published:** 2015-01-29

**Authors:** TOMOMI HIRATA, QINGFENG ZHENG, ZHAO CHEN, HIROYASU KINOSHITA, JUNICHI OKAMOTO, JOHANNES KRATZ, HUI LI, NATALIE LUI, HANH DO, TIFFANY CHENG, HSIN-HUI KATTY TSENG, KIYOSHI KOIZUMI, KAZUO SHIMIZU, HAI-MENG ZHOU, DAVID JABLONS, BIAO HE

**Affiliations:** 1 Thoracic Oncology Program, Department of Surgery, University of California, San Francisco, CA 94115, USA; 2 Department of Surgery, Division of Thoracic Surgery, Nippon Medical School, Tokyo 113-8602, Japan; 3 Key Laboratory of Carcinogenesis and Translational Research (Ministry of Education), Thoracic Surgery II, Peking University Cancer Hospital and Institute, Beijing 100142, P.R. China; 4 School of Life Sciences, Tsinghua University, Beijing 100084, P.R. China; 5 Helen Diller Family Comprehensive Cancer Center, University of California, San Francisco, CA 94143, USA

**Keywords:** mesothelioma, marker, chemotherapy

## Abstract

Malignant pleural mesothelioma (MPM) is a highly aggressive tumor that has a poor prognosis, limited treatment options, and a worldwide incidence that is expected to increase in the next decade. We evaluated *Wnt7A* expression in 50 surgically resected tumor specimens using quantitative PCR. The expression values, were assessed by clinicopathological factors and K-M and Cox’s regression with OS. The mean level of *Wnt7A* expression had a significant correlation with International Mesothelioma Interest Group (IMIG) stage (P<0.034), gender, smoking history and ethnicity, respectively (P=0.020, P=0.014, P=0.039). In the univariate analysis, low *Wnt7A* expression was a significant negative factor for overall survival (P=0.043, HR=2.30). However, multivariate Cox’s regression revealed no significant factors for overall survival (low *Wnt7A*: P=0.051, HR=2.283; non-epithelioid subtype: P=0.050, HR=2.898). In patients with epithelioid tumors, those with low *Wnt7A* expression had significantly worse prognosis (P=0.019, HR=2.98). In patients with epithelioid tumors, females had significantly better prognosis than males (P=0.035). In patients who did not have neoadjuvant chemotherapy, prognosis was significantly more favorable for patients with high *Wnt7A* expression than for those with low *Wnt7A* expression (P=0.031). Among the patients with low *Wnt7A-*expressing tumors, those who received neoadjuvant chemotherapy had better prognosis than those who did not (P=0.024). The results of our study suggest that *Wnt7A* expression is a putative prognostic factor and a predictor of chemosensitivity.

## Introduction

Malignant pleural mesothelioma (MPM) is an asbestos-induced, highly aggressive tumor that was once considered rare, but its incidence has steadily increased and new cases are predicted to peak between 2015 and 2025 (1,2). The disease is almost always fatal; median survival is 9 months for patients treated with supportive care, 12.1 months for those who receive the best available chemotherapy (3), and 11.7–13 months for those who have maximal cytoreductive surgery, as well as chemotherapy and/or radiation (4,5). The treatment with chemotherapy has shown slightly improved survival, and multi-modal trials are ongoing (1). A previous study has revealed that pemetrexed plus cisplatin neoadjuvant chemotherapy followed by extrapleural pneumonectomy (EPP) and hemithoracic radiation improved the 2-year overall survival rate (6).

Known positive prognostic factors for MPM include good performance status, young age, female, early stage and epithelioid subtype (7–10). Several markers have been proposed either as positive (e.g. Syndecan 1 expression) (11,12) or negative (e.g. Cox-2 expression) (13), and new microarray technology currently reveals batteries of genes that may have prognostic value (14,15). Although the two principal tumor suppressor genes, *Rb* (16–18) and *p53* (19), are not commonly absent in MPM, other molecules that are important in the *Rb* and *p53* pathways are present in MPM (1), particularly *p16* and *p14* (20,21). However, whether tumor suppressor expression is correlated with MPM prognosis is not known. Members of the Wnt signaling family are highly conserved and secrete glycoproteins vital to mammalian development and many aspects of adult tissue homeostasis. The members of this family regulate important cellular processes, such as proliferation, differentiation and cell fate specification (22). Furthermore, the Wnt gene family encodes transcription factors that regulate morphogenesis and cell differentiation during embryogenesis by activating or repressing the expression of target genes (23). Wnt signals are transduced through 7 transmembrane-type Wnt receptors encoded by Frizzled (Fz) genes to the β-catenin-TCF pathway, the JNK pathway or the Ca^2+^-releasing pathway. E-cadherin induction by Wnt/β-catenin signaling is an evolutionarily conserved pathway operative in lung cancer cells, and loss of *Wnt7A* expression may be important in lung cancer development or progression due to its effects on E-cadherin (24). Therefore, *Wnt7A* appears to play important roles in embryonic development and tumorigenesis (25–27). Wnt signaling molecules are potent targets for cancer diagnosis (susceptibility, metastasis and prognosis), cancer prevention and treatment, and for regenerative medicine or tissue engineering (28).

We therefore analyzed *Wnt7A* expression by using quantitative RT-PCR in surgically resected MPM specimens and/or adjacent normal tissues. We also examined the correlations between *Wnt7A* expression and various clinicopathological factors, prognosis and neoadjuvant chemotherapy.

## Materials and methods

### Patients

Between 1999 and 2005, 50 fresh samples of MPM and/or the adjacent normal tissues were collected from consecutive patients undergoing surgical resection in a study approved by the Committee on Human Research at the University of California, San Francisco (UCSF). Of these 50 patients, 24 had received neoadjuvant chemotherapy before surgery. The pathologic classification of each sample was confirmed by a review of sections stained with hematoxylin and eosin. Clinical information was also reviewed: age, gender, ethnicity, smoking status, Eastern Cooperative Oncology Group performance status (ECOG PS), histological subtype, International Mesothelioma Interest Group (IMIG) stage, surgical procedure, chemotherapy, radiation, recurrence status, vital status, progression status, and overall survival, which was calculated from the date of surgery.

### Tissues and RNA extraction

Tissue samples were promptly snap-frozen in liquid nitrogen and stored at −170°C before use. Total RNA was extracted using TRIzol LS (Invitrogen) and purified using the RNeasy Mini kit (Qiagen).

### Quantitative real-time reverse transcription-PCR

cDNA synthesis and Taqman PCR were performed as previously described (29). Hybridization probes and primers (*Wnt7A*, Hs01114990 m1; GAPDH, Hs00244574 m1) were purchased from Applied Biosystems (ABI). *Wnt7A* expression was assayed in triplicate using an ABI 7300 real-time PCR system. Samples were normalized to the housekeeping gene *GAPDH*, and expression levels were calculated using the 2^−ΔΔCt^ method compared to total RNA of the mixed adjacent normal pleural tissues derived from 11 patients.

### Statistical analysis

Patients were divided into those with high *Wnt7A* expression and those with low *Wnt7A* expression. These groups were compared with respect to the clinicopathological factors by using t-tests, Chi-square tests or Kaplan-Meier survival curves and the Log-rank test. A correlation between *Wnt7A* expression and IMIG staging system [stage, primary site (T) or lymph node metastasis (N)] was analyzed by one-way ANOVA. Kaplan-Meier survival curves for overall survival were compared for various clinicopathologic factors. Prognostic variables that were significant on univariate analysis were entered into a Cox’s proportional hazards model to determine the hazard ratio (HR). The cutoff values were associated with the lowest P obtained when comparing the two *Wnt7A* expression groups (the optimal P approach). Two-sided P-values <0.05 were considered significant. All analyses were conducted using the IBM SPSS statistics software package, ver. 18.0.

## Results

### Patient characteristics

Table I summarizes the characteristics of the 50 patients and prognosis after surgery according to *Wnt7A* expression (high *Wnt7A*, n=30; low *Wnt7A*, n=20). Performance status data were missing for 26 patients. The *Wnt7A* expression groups differed significantly in regards to gender, smoking status, performance status and vital status.

### Univariate analyses

In the univariate analysis for overall survival, gender, performance status, subtype and *Wnt7A* expression were all statistically significant (Table II). Worse overall survival correlated with low *Wnt7A* expression when expression was assessed as a categorical variable [≥4.315 (log_10_ (*Wnt7A* expression) = 0.635, P=0.043; HR=2.30 (95%CI, 1.01–5.25)].

### Multivariate analysis

Multivariate analysis using a Cox’s regression hazard model showed no significant prognostic factors for overall survival, but did show that low *Wnt7A* expression (P=0.051, HR=2.283) and histological subtype (non-epithelioid, P=0.05, HR=2.898) were likely to be negative prognostic factors for overall survival (Table III).

### Wnt7A expression and its relationship with clinicopathological characteristics and overall survival

**Figure 1:**
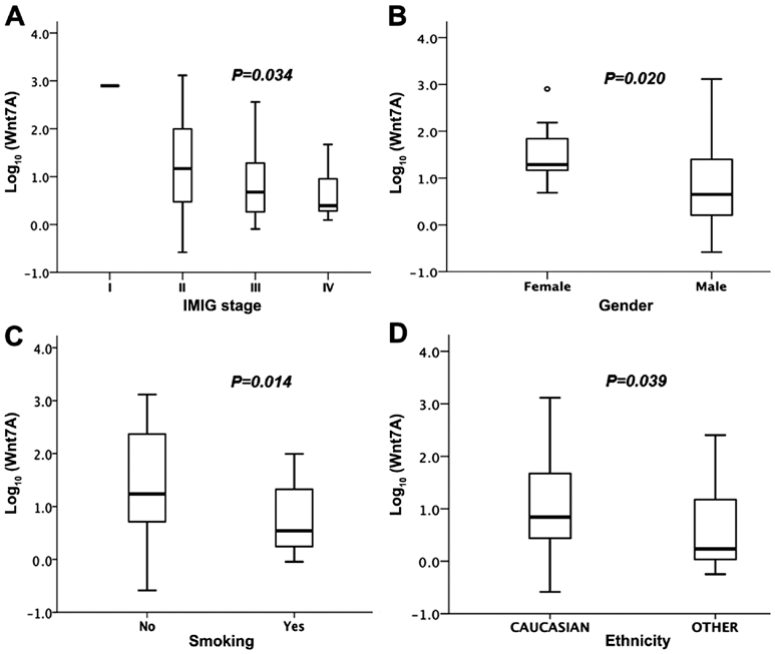
*Wnt7A* expression logarithm according to clinicopathological factors. (A) IMIG stage (I, II, III, IV), (B) gender, (C) smoking status and (D) ethnicity (Caucasian vs. other ethnicities). P-values were calculated by ANOVA in A and by 2-sided exact test in B–D, respectively.

*Wnt7A* expression differed significantly between the 11 paired normal and tumor tissues (P=0.005, Fig. 5), and by tumor stage (Fig. 1A), gender (Fig. 1B), smoking status (Fig. 1C) and ethnicity (Fig. 1D). The logarithm of *Wnt7A* expression values in the tumors ranged from −0.58 to +0.89 (mean ± SD, 0.98±0.14). The frequency distribution graph showed two peaks (Fig. 6), which allowed two subgroups, high-risk (low *Wnt7A*) and low risk (high *Wnt7A*) to be defined. A cutoff point for distribution of *Wnt7A* expression into 2 groups was optimized at 4.67 in the logarithm of *Wnt7A* expression to acquire a minimum P-value in the difference of overall survival between the 2 groups.

**Figure 2:**
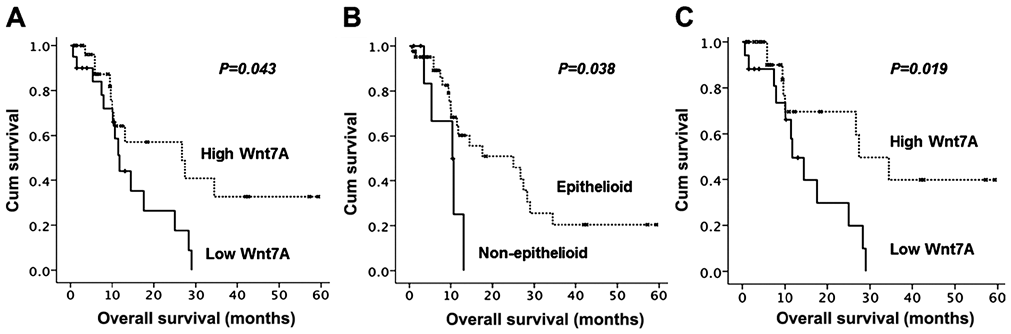
Kaplan-Meier survival curves stratified by *Wnt7A* expression. (A) Overall survival of all patients according to *Wnt7A* expression (n=50). (B) Overall survival of all patients according to histological subtype (epithelioid vs. non-epithelioid). (C) Overall survival of patients with epithelioid tumors according to *Wnt7A* expression.

Overall survival was significantly more favorable for patients with high *Wnt7A* expression than for those with low expression (26.7±11.1 months vs. 11.8±4.9; P=0.043, Fig. 2A). To confirm a correlation between *Wnt7A* expression and survival, we divided the distribution of *Wnt7A* expression into thirds (low *Wnt7A*, intermediate *Wnt7A*, high *Wnt7A*) using the 33rd and 66th percentiles. We found that Kaplan-Meier curves plotted for epithelioid tumors showed better prognosis (P=0.023, Fig. 6). Previously Fennel *et al* estimated in a similar way (30).

Overall survival was also significantly more favorable for patients with epithelioid tumors than for those with non-epithelioid tumors (P=0.038). In the 42 patients who had epithelioid tumors, overall survival was significantly worse when the tumors had low *Wnt7A* expression (Fig. 1C). MST of high *Wnt7A* was 25±5.2 (95% CI, 8.8 to 41.2) months, whereas MST of low *Wnt7A* was 10.4±2.6 (95% CI, 5.2 to 15.6) months.

**Figure 3:**
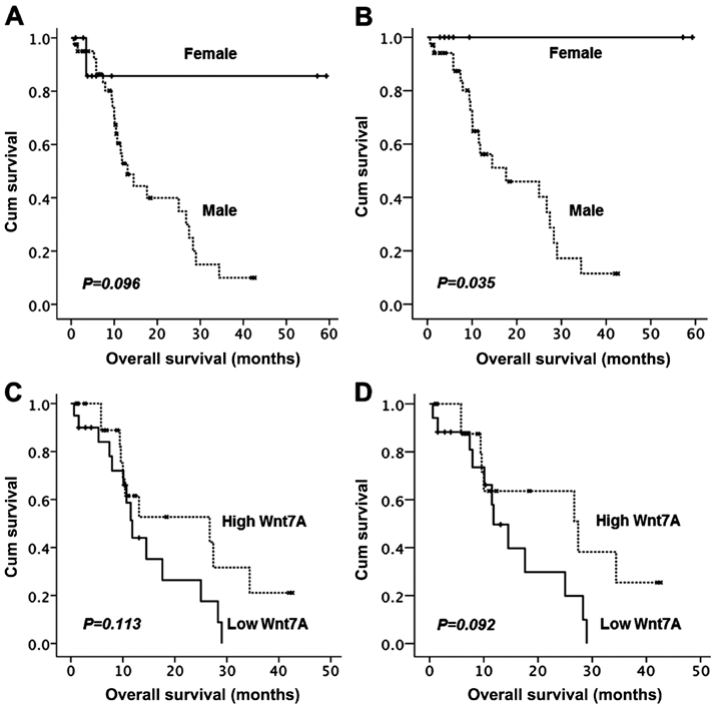
Kaplan-Meier survival curves according to gender and *Wnt7A* expression. (A) Overall survival of all patients (n=50) according to gender. (B) Overall survival of patients with epithelioid tumors (n=42) according to gender. (C) Overall survival of male patients (n=41) according to *Wnt7A* expression. (D) Overall survival of male patients with epithelioid tumors (n=35) according to *Wnt7A* expression.

All 9 female patients with MPM had high *Wnt7A* expression. They also had a more favorable overall survival than the male patients (P=0.096, Fig. 3A), including the subgroup with epithelioid tumors (P=0.034, Fig. 3B). Among the men with epithelioid tumors, those with high *Wnt7A* expression had better survival than those with low *Wnt7A* expression (P=0.092, Fig. 3D).

**Figure 4:**
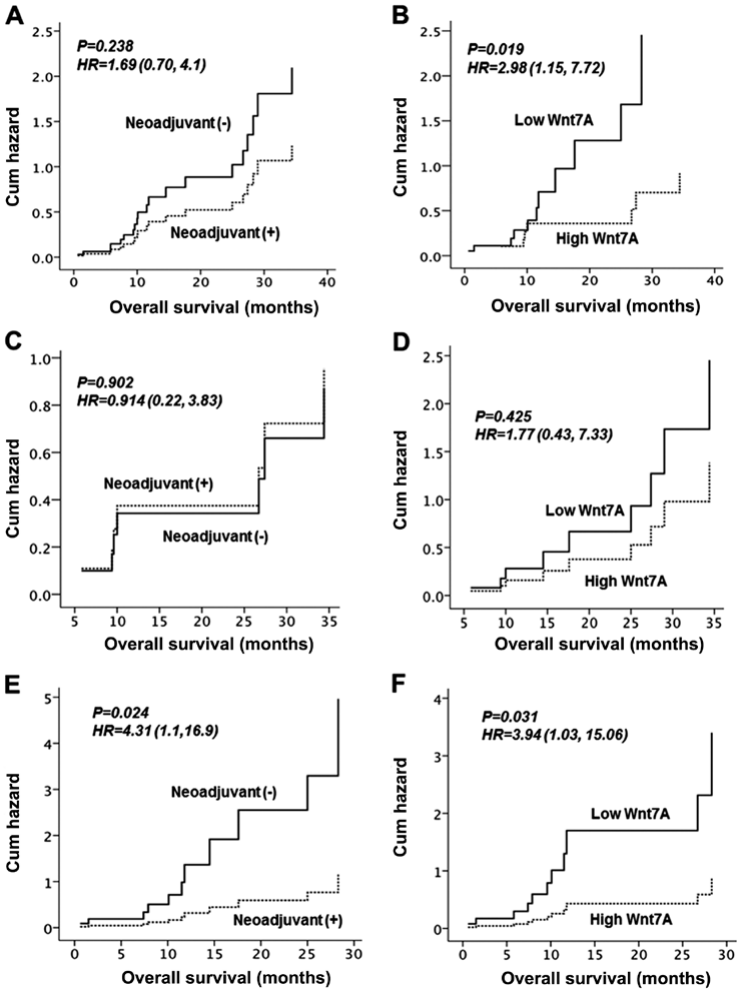
Hazard curves stratified by neoadjuvant chemotherapy and *Wnt7A* expression. Overall survival according to (A) neoadjuvant chemotherapy, (B) Wnt7A expression. (C) Overall survival of patients with high *Wnt7A* tumors according to neoadjuvant chemotherapy. (D) Overall survival of patients with neoadjuvant chemotherapy according to Wnt7A expression. (E) Overall survival of patients with low *Wnt7A* tumors according to neoadjuvant chemotherapy. (F) Overall survival of patients without neoadjuvant chemotherapy according to Wnt7A expression.

**Figure 5:**
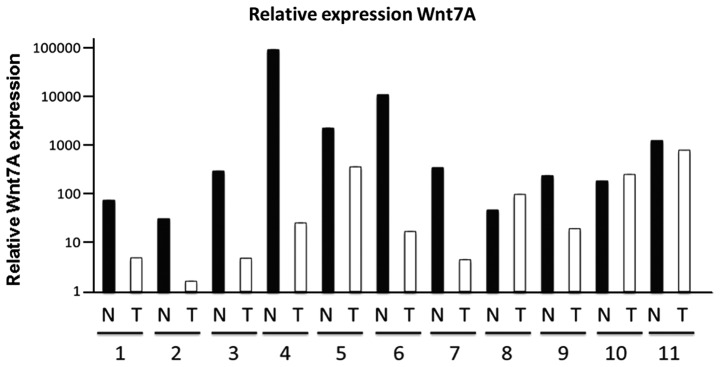
*Wnt7A* expression differed significantly between the 11 paired normal (N) and tumor tissues (T).

**Figure 6:**
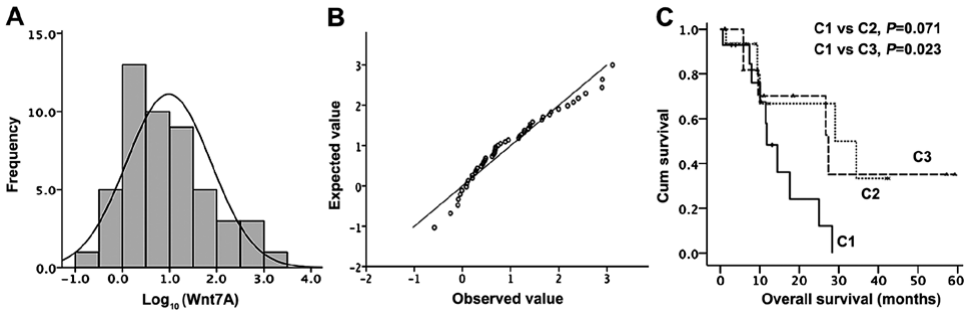
Statistical analysis of *Wnt7A* expression. (A) Frequency distribution of *Wnt7A* expression value logarithm. (B) Normal probability plot showing conformity of a normal distribution. (C) The Kaplan-Meier curves plotted for epithelioid tumors showed better prognosis (C1, low *Wnt7A*; C2, intermediate *Wnt7A*; C3, high *Wnt7A*; C1 vs C2, P=0.071; C1 vs C3, P=0.023).

Survival analysis of the 42 patients with epithelioid tumors showed that overall survival for the 20 patients who received neoadjuvant chemotherapy was not significantly better than that for the 22 patients who did not (Fig. 4A). Overall survival was significantly better for patients with high *Wnt7A-*expressing epithelioid tumors than for those with low *Wnt7A-*expressing tumors (27.4±0 vs. 10.1±1.7 vs. 10.1±1.7; 95% CI, 6.6 to 13.6; P=0.019, Fig. 4B). In the patients with high *Wnt7A-*expressing epithelioid tumors, overall survival did not differ between those who underwent neoadjuvant chemotherapy and those who did not (P=0.902, Fig. 4C). However, in patients with low *Wnt7A-*expressing epithelioid tumors, overall survival was significantly better in those who underwent neoadjuvant chemotherapy than in those who did not (P=0.024; HR=4.31, 95% CI of HR, 1.1 to 16.9; Fig. 4E). In the subset of the 20 patients who received neoadjuvant chemotherapy, overall survival did not differ significantly between those with low *Wnt7A* vs. high *Wnt7A* tumors (17.6±5.25; 95% CI, 7.3 to 27.9 months vs. 34.4±13.3; 95% CI, 8.2 to 60.5 months, P=0.425; Fig. 4D). In the subset of 22 patients who did not have neoadjuvant chemotherapy, overall survival was significantly better for patients with high *Wnt7A-*expressing tumors than for those with low *Wnt7A*-expressing tumors (P=0.031; 95% HR=1.03 to 15.06; Fig. 4F).

## Discussion

The Wnt genes compose a large gene family encoding a group of secreted signaling molecules that have been implicated in oncogenesis and a number of developmental processes. Expression of *Wnt7A* is restricted to certain tissues: placenta, kidney, testis, uterus, fetal lung and fetal and adult brain. Why study it in MPM? Our PCR analysis of *Wnt7A* expression in MPM showed that low *Wnt7A* expression (P=0.051, HR=2.283) and histological subtype (non-epithelioid, P=0.05, HR=2.898) were likely to be negative prognostic factors for overall survival. Our survival analyses indicated that *Wnt7A* expression was correlated with overall survival in the univariate analysis, but not in the multivariate Cox’s regression.

In our study, *Wnt7A* expression was significantly higher in women with MPM than in men (Fig. 1B), and gender was a positive prognostic factor in patients with epithelioid tumors (Fig. 3B). *Wnt7A* is required for proper differentiation and gland formation during uterine development. Following post-natal growth, *Wnt7A* expression becomes restricted primarily to the luminal epithelium and is responsible for maintaining expression of other Wnts in the stroma (31–33). The *Wnt7A* gene is known to guide the development of the anterior-posterior axis in the female reproductive tract, and to play a critical role in uterine smooth muscle patterning and maintenance of adult uterine function. This gene is also responsive to changes in the levels of sex steroid hormone in the female reproductive tract. An inverse association for mRNA expression was found between *Wnt7A* and estrogen receptor α (ER-α) (34). Hypersensitivity of leiomyoma cells to estrogen may deregulate *Wnt7A* expression (34). Decreased *Wnt7A* expression may lead to loss of control in the patterning of the myometrium and result in the development of leiomyoma (34). In particular, the recent finding that ER-β acts as a tumor suppressor has great potential relevance to predicting disease progression and therapeutic response in patients with MPM (35).

E-cadherin induction by Wnt/β-catenin signaling is an evolutionarily conserved pathway operative in lung cancer cells, and loss of *Wnt7A* expression may be important in lung cancer development or progression through its effects on E-cadherin. Apparent physiologic levels of *Wnt7A* positively regulate E-cadherin expression in lung cancer (24).

During development, the Wnt pathway affects cell fate, polarity and proliferation, and *Wnt7A* has been implicated in the maintenance of HOX expression. In contrast to what occurs in normal lung and mortal short-term bronchial epithelial cultures, *Wnt7A* was frequently less expressed or absent in lung cancers. It is possible that those genes that are normally expressed in undifferentiated cells are upregulated in cancer, similar with other Homeobox genes (23,36).

In our study, *Wnt7A* expression was highly correlated with IMIG stage (Fig. 1A). The primary tumor (T) seemed to contribute to stage than lymph node metastasis (N) (data not shown). These results indicate that the role of *Wnt7A* is to have cells differentiated and kept normal or restored to original specific morphology of the organ, and if it is lost or decreased, it may have consequence for worse prognosis in MPM.

In our study, *Wnt7A* expression might have been affected by smoking in normal or precancerous events such K-ras mutation, loss of EGFR mutation, or P53 mutation, even in pleural tissues. We found that MPM patients with a smoking history had lower *Wnt7A* expression than those who did not (Fig. 1C). Smoking considerably increases the risk of developing mesothelioma. A smoker who is exposed to asbestos has a 50- to 90-fold greater chance of developing mesothelioma whereas a non-smoker exposed to asbestos has a 5-fold greater chance (37,38). It is possible that smoking might decrease *Wnt7A* expression associated with tumor-associated macrophages (39).

The results of our univariate analysis showed that gender, Eastern Cooperative Oncology Group performance status (ECOG PS), surgical procedure, and *Wnt7A* expression were not significant predictors of overall survival. With the exception of performance status, these findings are identical to those reported previously (7–10). ECOG PS, usually one of the most reliable prognostic factors, was not a significant predictor of survival in our study. However, ECOG PS data were not available for more than half of our patients, so we could not compare the relative contribution of ECOG PS to prognosis with that of *Wnt7A* expression. We did find that *Wnt7A* expression and histological subtype were similarly prognostic in the Cox’s model, indicating that they could be prognostic markers for MPM.

The use of neoadjuvant chemotherapy before surgery for MPM has recently become common. However, neoadjuvant chemotherapy remains investigational because it has not definitively showed a survival benefit (40–42). A recent multicenter phase II trial using pemetrexed plus cisplatin neoadjuvant chemotherapy followed by extrapleural pneumonectomy (EPP) and hemithoracic radiation demonstrated feasibility and a reasonable long-term survival rate (6). On the other hand, neoadjuvant chemotherapy appeared to affect compliance with surgery and radiation therapy. These findings point to the need to determine which patients will benefit from neoadjuvant chemotherapy before surgery. The European Organization for Research and Treatment of Cancer (EORTC) prognostic score (EPS) for MPM, based on three consecutive phase II trials using different chemotherapy agents, found no association between objective tumor response and EPS classification (30). In our study, the survival analysis of 42 patients with epithelioid tumors showed that overall survival for the 20 patients who received neoadjuvant chemotherapy was not significantly better than that for 22 patients who did not. In the patients with high *Wnt7A* epithelioid tumors, overall survival did not differ between those who underwent neoadjuvant chemotherapy and those who did not (P=0.902, Fig. 4C) while in patients with low *Wnt7A* epithelioid tumors, overall survival was significantly better in those who underwent neoadjuvant chemotherapy than in those who did not. These results indicate the patients with low *Wnt7A* expression do benefit, while patients with high *Wnt7A* expression do not benefit. Thus, we recommend neoadjuvant chemotherapy for patients with low *Wnt7A* tumors.

Among patients who did not have neoadjuvant chemotherapy, those with high *Wnt7A* expression had a significantly better prognosis than those with low *Wnt7A* expression. However, among patients who did have neoadjuvant chemotherapy, those with low *Wnt7A* expression had a significantly better prognosis than those with high *Wnt7A* expression. This indicates that *Wnt7A* is a putative novel prognostic factor for MPM and a novel predictor for determining whether neoadjuvant chemotherapy will be beneficial for patients with MPM. This means that patients with high *Wnt7A* expression have better prognosis, and patients with high *Wnt7A* expression might be more sensitive to neoadjuvant chemotherapy and they could have a better prognosis than those without neoadjuvant chemotherapy.

In conclusion, our study revealed that *Wnt7A* is a putative novel prognostic factor for MPM and a novel predictor for determining whether neoadjuvant chemotherapy will be beneficial for patients with MPM. Finally, our results suggest that *Wnt7A* is possibly a novel tumor-suppressor gene in MPM.

## Figures and Tables

**Table I:** Clinicopathological characteristics of the patients with malignant pleural mesothelioma, according to *Wnt7A* expression.

	Total (n=50)	High *Wnt7A* (n=30)	Low *Wnt7A* (n=20)	P-value
Age (years)				
Mean ± SD	66±9	67±10	67±9	0.942
Range	(45–84)	(59–84)	(54–84)	
Gender				
Male/female	41/9	21/9	20/0	0.007
Ethnicity				
Caucasian/other	38/12	25/5	13/7	0.182
Smoking				
Yes/no	16/24	14/11	2/13	0.024
ECOG PS				
0/1/2/ND	10/13/1/26	6/4/0/20	4/9/1/6	0.026
Subtype				
Epithelioid/other	42/8	25/5	17/3	0.875
IMIG stage				
I/II/III/IV	1/18/24/5	1/13/13/2	0/5/11/3	0.363
Primary tumor (T)				
T1/T2/T3/T4	1/20/22/5	1/16/11/3	0/4/11/2	0.550
Regional lymph node (N)				
N0/N1/N2	37/5/6	24/1/4	13/4/2	0.293
Surgical procedure				
EPP/PLE/CWR/BIO	11/35/2/2	8/19/2/1	3/16/0/1	0.451
Chemotherapy				
Yes/no	30/20	18/12	12/8	1.000
Neo/PSC/both	24/8/2	15/4/1	11/4/1	0.529
Neo, yes/no	24/26	15/15	9/11	0.447
PSC, yes/no	8/42	4/26	4/16	0.351
Radiation				
Yes/no	20/30	12/18	8/12	0.162
Progression				
Yes/no	19/31	17/13	12/8	0.525
Vital status				
Alive/deceased	25/25	19/11	6/14	0.042
Overall survival (OS)				
Median OS time	14.5±4.5	26.7±11.2	11.8±1.0	0.043

ECOG PS, Eastern Cooperative Oncology Group performance status; ND, not defined; IMIG, International Mesothelioma Interest Group; EPP, extrapleural pneumonectomy; PLE, pleurectomy; CWR, chest wall resection; BIO, biopsy; Neo, neoadjuvant chemotherapy; PSC, postsurgical chemotherapy.

**Table II:** Univariate analysis of overall survival in patients with malignant pleural mesothelioma.

	Overall survival		
	
	P-value	Hazard ratio	95% CI
Age (average, 66±9)			
≤66/>66 years	0.347	1/1.47	(0.66–3.29)
Gender			
Male/female	0.096	1/0.46	(0.17–1.26)
Ethnicity			
Caucasian/other	0.242	1/1.63	(0.71–3.71)
Smoking			
No/yes	0.163	1/1.99	(0.74–5.40)
ECOG PS			
0/1+2	0.01	1/3.89	(1.29–11.8)
Surgical procedure			
EPP/PLE/CWR/BIO	0.056	0/2.44/0/1	PLE (0.33–18.32)
IMIG staging system			
1+2/3+4	0.181	1/1.754	(0.76–4.04)
Primary tumor (T)			
T1+T2/T3+T4	0.241	1/1.63	(0.72–3.68)
Regional lymph node			
N^−^/N^+^	0.816	1/1.13	(0.382–3.390)
Subtype			
Epithelioid/other	0.038	1/2.91	(1.01–3.34)
Neoadjuvant chemotherapy			
Yes/no	0.423	1/1.14	(0.63–3.03)
Adjuvant chemotherapy			
Yes/no	0.143	1/0.47	(0.17–1.32)
Radiation therapy			
Yes/no	0.395	1/0.71	(0.32–1.58)
*Wnt7A* expression			
High/low	0.043	1/2.30	(1.01–5.25)

CI, confidence interval; ECOG PS, Eastern Cooperative Oncology Group performance status; EPP, extrapleural pneumonectomy; PLE, pleurectomy; CWR, chest wall resection; BIO, biopsy; IMIG, International Mesothelioma Interest Group; N^−^, lymph node metastasis-negative; N^+^, lymph node metastasis-positive.

**Table III:** Results of the multivariate analysis Cox’s regression hazard model for overall and progression-free survival.

						95% CI for HR	
						
	β	SE of β	Wald	P-value	HR	Lower	Upper
Non-epithelioid	1.064	0.544	3.827	0.05	2.898	0.998	8.416
Low Wnt7A	0.825	0.422	3.824	0.051	2.283	0.998	5.22

95% CI, 95% confidence interval; HR, hazard ratio; SE, standard error; Wald, Wald statistic for logistic regression algorithms. ECOG PS was not included in this multivariate analysis.
